# Resilience and coping strategies in relation to mental health outcomes in people with cancer

**DOI:** 10.1371/journal.pone.0252075

**Published:** 2021-05-24

**Authors:** Patricia Macía, Mercedes Barranco, Susana Gorbeña, Esther Álvarez-Fuentes, Ioseba Iraurgi

**Affiliations:** 1 Department of Personality, Evaluation and Psychological Treatments, Faculty of Psychology and Education, University of Deusto, Bilbao, Biscay, Spain; 2 Provincial Office of Biscay, Spanish Association Against Cancer, Bilbao, Spain; Universite de Bretagne Occidentale, FRANCE

## Abstract

Considering the importance of psychological variables on health-related processes, this study investigated the role of resilience and coping strategies in relation to health. The aim of this research was to explore the underlying association between these aspects for the better understanding of the effect of psychosocial variables on mental health in cancer. This information could lead to the design of adapted psychological interventions in cancer. Participants with different diagnosis of cancer were recruited (*N* = 170). They came from the Spanish Association Against Cancer of Biscay. Resilience was measured with the 10 items Connor-Davidson Resilience Scale, coping with the Cognitive Emotion Regulation Questionnaire and mental health was measured as a global indicator through the SF-12 and the GHQ-12. A structural equation model (SEM) was conducted to test the effects between the constructs. Results showed that resilience and coping were significantly associated. Results reflected an absence of significant correlation between adaptive and disadaptive coping strategies. Resilience was the factor that most correlated with health outcomes (*β* = –.45, p < .001). However, disadaptive coping strategies did not correlate with resilience or mental health indicators. Findings in this study underscore the positive contribution of high levels of resilience and an adaptive coping on participants´ level of health. Disadaptive coping strategies did not reflect any positive relation with resilience or health indicators. Thus, promoting resilience and adaptive coping could be a significant goal for psychosocial and educational interventions in people with cancer.

## Introduction

The diagnosis and treatment of cancer are considered stressful life experiences that usually result in emotional disturbances [1, 2]. In many cases, cancer patients experience high levels of emotional distress, including symptoms of anxiety, depression, stress, etc., although only few people will develop long-term psychological severe disorders in reaction to this stressful event [3–5]. Nevertheless, the literature also suggests that individuals have the capacity to cope and adapt to highly stressful situations [6]. In this sense, some resources such as coping strategies and seeking social support have been considered adequate means to face these types of events [7].

The concept of coping consists of behaviours, actions and thoughts that enable individuals to deal with the demands of events that are conceived as stressful. Lazarus and Folkman [8] classified coping strategies into two different types: problem-focused and emotion-focused coping. Problem-focused coping aims to manage or modify the problem that is generating discomfort, dealing with the stressor in different ways, such as planning actions or seeking information. On the contrary, emotion-focused coping attempts to regulate the emotional response to the problem, through methods like looking for support [9]. These types of coping strategies usually involve different adaptive strategies that enable individuals to cope life situations, and that are used to the extent that they are more or less adaptive at any given time [10]. However, it is also important to analyse which kind of strategies can be disadaptive, with the aim of identifying those which can increase risk of maladaptive health behaviours and higher psychological distress [11]. Literature suggests that in cancer population certain strategies are more widely used and adaptive than others. Adaptive coping usually can benefit individuals suffering the disease, as it leads to more constructive or positive coping processes. Conversely, disadaptive coping strategies are considered as more dysfunctional or negative [9].

As it can be expected, the use of a certain type of coping or other is important considering the characteristics of cancer disease, which can be a life-threating illness that lead patients to have to make decisions about their own-health rapidly, for instance, about their oncological treatment and other aspects related to the disease. The use of particular coping strategies has an impact on patients´ perception about their illness, and consequently, affect their level of mental health and quality of life [11]. It has been widely recognized that coping helps adapting to change, which is important in achieving or maintaining psychological well-being [10]. In fact, some authors have found that coping through acceptance and seeking emotional support correlated with higher quality of life and general mood, while using disadaptive coping strategies such as self-blame and negation correlated negatively with mental health outcomes [12, 13]. Further, cognitive strategies based on planning actions, accepting life-events, positive revaluation or adopting a humorous approach revealed improved mental health outcomes in cancer patients undergoing oncological treatment [1]. These patients showed positive changes in personal skills and resources for facing cancer disease, as well as lower anxiety and depression levels one year after diagnosis, related to a better psychological quality of life. On the other hand, social support seeking was not related to better mental health levels in initial phases of the disease, but is was significantly correlated with improved mental health outcomes during a period of adjuvant treatments [1]. Findings emphasize the significance of promoting adaptive and effective coping ways for the improvement of psychological adjustment in cancer patients [12, 13].

Another concept closely related to adjustment to change is resilience. It has been defined as the competence or effective coping in response to stressful or traumatic events, which involves risk or adversity [14, 15]. What characterizes resilient people is the ability to emerge even stronger from the adverse situation, being able to improve their coping strategies and raising their levels of adaptation and well-being [10]. Resilient people may present protective personal attributes that enable adaptation to cancer [14], including cognitive flexibility, positive emotions and active coping [16].

Some investigations in this area have integrated variables of coping and resilience in order to explore their relationship and influence on individual´s mental health outcomes. Mayordomo et al. [10] proposed a model based on the effects of resilience and coping strategies on adults´ well-being in a normative sample (*n* = 305). They found evidence about the prediction of psychological well-being through a confirmatory model, which showed good fit indices. The results showed that psychological well-being was positively predicted by problem-focused coping and resilience, whereas emotion-focused coping had a negative influence on well-being. Furthermore, Tomás et al. [17] investigated the role of resilience and coping as predictors of well-being in a Spanish sample of 225 elderly people, through a structural model. Results showed that resilience was a significant predictor of the variance in well-being, in contrast to coping strategies.

Besides, most studies have found that the efficacy of any given coping strategy may also be related to personal resilience. Some authors have found that higher resilience was related to greater use of an active and minimising coping styles, which leaded to lower levels of negative affect or depressive symptoms, lower level of anxiety and a better sleep quality [18]. High levels of resilience have demonstrated to be linked to better emotional accommodation in cancer patients, what suggests that resilience might be a protective factor against emotional discomfort [19, 20]. Ye et al. [21] also found that resilience had a mediating role in the relation between stress, negative affect and quality of life. In fact, cancer patients with high resilience showed higher adaptive functioning [4, 19].

Considering the importance of psychological variables on health-related processes (regarding levels of quality of life, well-being, adherence to treatment…) [10], variables such as resilience and coping strategies have been explored. However, it is necessary to go deeper into such variables in cancer samples, since knowing the underlying functioning of these variables would allow the design and guidance of therapeutic actions with greater precision. The aim of this study was precisely to analyse the relationship between resilience, coping strategies and health, conceiving health as a general component including aspects related to mental health outcomes and quality of life. We aimed to explore the underlying association between these aspects for the better understanding of the effect of psychosocial variables on health status (as an outcome or dependant variable) in people with cancer.

## Materials and methods

### Ethics

This study was carried out in accordance with the recommendations of the Helsinki declaration and the guidelines of the International Committee of Medical Journal Editors. All subjects gave written informed consent in accordance with the Declaration of Helsinki. The protocol was approved by the University of Deusto Research Ethics Committee (ETK-19/19-20).

### Participants

Participants being diagnosed for different types of cancer (*n* = 170) and almost all of them (92.9%) undergoing oncological treatment (chemotherapy, radiotherapy…) were recruited from April 2019 to April 2020. They came from the Spanish Association Against Cancer (AECC) of Biscay, where they were attended for supporting and/or counselling services provided by the psychotherapeutic team of the association. Inclusion criteria were: to be over 18 years old, to suffer or have suffered from cancer diagnosis, and to be contact of the AECC. On the contrary, exclusion criteria were to be under 18 years old and to have never been diagnosed with cancer.

### Procedure

Psychologists from the Spanish Association Against Cancer of Biscay with ample experience in cancer patient care collected data. All the participants who were attending the association, or at least had a close relation with it, were asked to participate voluntarily in the study. They were provided with information about the study by email or at the premises of the association. In case affirmative, a written informed consent was obtained before data collection to meet the ethical and legal requirements of the research project. Each individual completed the self-administered questionnaires (see Instruments), which could be answered in paper (in the association) or online, as best suited them. Completing the questionnaire took them about 50 minutes, approximately. If any emotional reactions emerged, the psychologists of the AECC committed to maintain an empathic attitude, providing support.

### Instruments

A self-administered questionnaire was used to collect information, including socio-demographical and clinical data related to the disease. Four psychometric instruments were used to explore the variables of interest: resilience, adaptive and disadaptive coping strategies, mental health and quality of life in people with cancer.

#### Resilience

Resilience was measured with the 10 items Connor—Davidson Resilience Scale [22]—CD-RISC was used [23]. The 10 items were rated using a 5-point Likert scale, with a response format ranging from “1 = totally disagree” to “5 = totally agree”. The total level of resilience was given by the sum of the total items, so that higher scores indicate higher level of resilience. The instrument showed good psychometric qualities. Cronbach´s alpha in the original study was.85, and.81 in the Spanish version of 10 items [24]. Cronbach´s alpha for this study was.91, showing also good psychometric qualities.

#### Coping strategies

In order to assess coping strategies, the Cognitive Emotion Regulation Questionnaire scale (CERQ- Cognitive Emotion Regulation Questionnaire-Short) was selected [25]. The scale was developed to evaluate the cognitive assessment of the person when facing adverse and stressful life events. The original scale of 36 items was divided into nine subscales that were conceptually group in two main dimensions or coping strategies: adaptive and disadaptive coping. The instrument was also adapted to Spanish version [26] and for this study, the shorter version of 18 items was used and it has the same dimensional structure. Each item was measured on a scale with five response options ranging from “1 = hardly ever or never” to “5 = almost always”. The CERQ presents good psychometric qualities, with Cronbach´s alpha coefficients over .80. Furthermore, it has shown a good factorial, discriminative and construct validity [25]. Cronbach´s alpha in the present study was analysed for each dimension: adaptive strategies (*α* = .85) [acceptance (*α* = .96), focusing on the planning (*α* = .74), positive refocusing (*α* = .82), positive revaluation (*α* = .82), putting the situation into perspective (*α* = .82),] and disadaptive strategies (*α* = .79) [self-blame (*α* = .84), blaming others (*α* = .83), rumination (*α* = .78) and catastrophism (*α* = .91)]. The internal consistency obtained in this study -for all the dimensions- showed Cronbach´s alpha value of.76.

#### Quality of life

Perception of quality of life was evaluated with the General Health Questionnaire SF-12 [27], which is based on the SF-36 [28]. It was adapted into Spanish version [29], showing good internal consistency levels over *α* = .70 in all the subscales. The questionnaire assesses eight main dimension of health: Physical Functioning (PF), Role limitations due to Physical health problems (RP), Social Functioning (SF), Bodily Pain (BP), Mental Health (MH), Role limitations due to Emotional problems (RE), Vitality (VI) and General Health (GH). The instrument presents good internal consistency, with a Cronbach´s alpha of.77. The overall score is obtained by summing the scores, evaluated through a Likert scale. It is measured in ascending order, so that a higher score means a higher perception of quality of life. Besides, the instrument provides two total scores or components (mental and physical health—TMC (*α* = .87), and TPC (*α* = .89) respectively), which are expressed in standardised T scores. These scores were obtained through the application of specific algorithms, which were provided by the group of people that adapted the instrument in Spain, under the direction of the Municipal Institute of Medical Research of Barcelona.

#### Mental health

Mental health in people with cancer was assessed with the General Health Questionnaire—GHQ-12 [30–32]. The GHQ-12 is a self-administered questionnaire developed with the purpose of detecting diagnosable psychiatric disorders. The questionnaire is intended for adults who have to respond indicating the frequency with which they have experienced some symptoms. In its 28-item version it measured four domains: somatic symptoms, anxiety and insomnia, social dysfunction and severe depression [30]. The shorter 12-items version evaluates two main aspects: the inability to develop basic and healthy functions and the presence of distressing phenomena. Each item was measured on a Likert scale with four response options ranging from “0 = better than usual” to “3 = much worse than usual”. The average sum of its items provides a scalar indicator of the degree of mental distress. The scale had good internal reliability, showing a Cronbach´s alpha for the 12-items Spanish version was *α* = .76, and *α* = .93 for this study.

### Statistical analyses

Descriptive statistics (means [*M*] and standard deviations [*SD*]) were calculated for variables of resilience, coping strategies (independent variables [IVs]) and mental health (dependent variable [DV]). All measures were transformed to a decimal scale to facilitate a better comprehension. Then, a correlational analysis was conducted to know the relationships between the variables of interest. Thirdly, a hierarchical regression model was performed in order to explore the specific influence of resilience and coping strategies (as principal variables) on each on the health´s indicators (as an outcome variable). For this, mental health as a global indicator, was analysed through three components separately: mental health (GHQ-12), mental component of quality of life (TMC) and physical component of quality of life (TPC). SPSS software version 22 was used to perform these statistical analyses [33].

Further, to conduct the statistical analysis a structural equation model (SEM) was performed to test the effects between the constructs, estimated with the EQS 6.1.27 [34]. The model was computed to test the relationships among different factors without measurement errors. An analysis of the measurement model would indicate if the observed variables measured the latent constructs. Adequate indexes in an initial estimation of the structural model would justify the existence of a conceptual relationship among the different dimensions. To assess the plausibility of the structural equation model, different fit criteria were used [35]: (a) the Chi-Square statistic; (b) a Comparative Fit Index (CFI) above.90; (c) the Goodness-of-Fit Index (GFI) as a measure of proportion of variance or covariance explained through the model, with adequate values above.90; (d) the Adjusted Goodness-of-Fit Index (AGFI) (above.90); the Standardized Root Mean Square Residual (SRMR); and (e) a Root Mean Squared Error of Approximation (RMSEA) (below.08). The model was computed assuming that each observed variable was significantly contributing to its respective latent variable.

## Results

### Sociodemographic data

Participants were adults with a range age between 20 to 82 years old (*M* = 49) and 78.8% of them were women (see Table 1). All of them had been diagnosed with different types of cancer [breast cancer (35.4%), lung (10.2%), colon (7.1%), gynaecological cancer (4%), prostate (3.9%), pancreas (2.7%), bladder (2.4), and others (34.3%)] and 48.5% of them were in an advanced stage of the disease (stages III and IV). Some of the individuals (almost 50%) had received other types of medical treatment besides the oncological one. With respect to sociodemographic variables, most of the participants were married (69.4%). Academically, 50% of them had a university degree and 21.2% had professional training. In the professional area, 47.6% of them were working, 6.5% were unemployed, 16.5% retired and 25.9% were in a situation of inability.

**Table 1:** Socio-demographic and clinical variables for the oncological sample.

Socio-demographic variables		Total		Clinical variables	Total	
		(*n* = 170)			(*n* = 170)	
		n	%		n	%
Gender (%)	Woman	134	78.8	Stages:		
	Man	36	21.2	I	15	8.8
Studies (%)	Primary school	18	10.6	II	19	11.2
	Secondary school	8	4.7	III	22	13.0
	Bachelor	21	12.4	IV	60	35.5
	Professional training	36	21.2	Oncological treatment:		
	University	85	50.0	Yes	158	92.9
	Others	2	1.2			
Employment (%)	Paid work	81	47.6	No	12	7.1
	Unpaid work	1	0.6	Other medical treatment:		
	Unemployed	11	6.5	Yes	84	49.4
	Retired	28	16.5	No	86	50.6
	Inability	44	25.9			
	Others	5	2.9			
Civil status (%)	Single	25	14.7			
	Married, in couple	118	69.4			
	Separated, divorced	19	11.2			
	Widower	5	2.9			
	Others	3	1.8			

**Note**. *n* = sample size.

### Descriptive statistics

Descriptive statistics were calculated for the main variables. Average scores were 6.26 (*SD* = 3.28) for resilience, 5.69 (*SD* = 2.08) for adaptive coping and 2.25 (*SD* = 1.62) for disadaptive coping, 4.47 (*SD* = 2.21) for mental health, 4.20 (*SD* = 1.07) for the mental component of quality of life and 4.15 (*SD* = 1.22) for the physical component of quality of life.

### Correlational analysis

A correlational analysis was conducted with the variables of interest (see Table 2). In general, all the variables were statistically and negatively associated with health, except from disadaptive coping strategies, which were significantly but positively correlated with health (*r* = .58, *p* < .001).

**Table 2:** Correlation analysis of variables or resilience and coping strategies with health.

		M	SD	*α*	1	2	3	4	5	6
	Health									
1	GHQ	4.47	2.21	.93	1					
2	SF-M	4.20	1.07	.87	-.74**	1				
3	SF-F	4.15	1.22	.89	-.42**	.07	1			
4	Resilience	6.26	3.28	.91	-.64**	.48**	.23**	1		
5	Adap cop	5.69	2.08	.85	-.26**	.23**	.02	.34**	1	
6	Disad cop	2.25	1.62	.79	.58**	-.56**	-.18*	-.44**	-.10	1

**Note**. M = mean; SD = standard deviation; *α* = Cronbach´s Alpha;GHQ = general health; SF-M = mental quality of life; SF-F = physical quality of life.

** = p < .001; p < .05.

### Hierarchic regression model

Table 3 presents the predictive model conducted through a hierarchical regression analysis. Firstly, resilience (*β* = –.45, *p* < .001) and disadaptive coping (*β* = .38, *p* < .001) (as predictor variables) showed a statistically significant correlation with mental health (output variable). However, no significant association was found between adaptive coping and mental health. Secondly, resilience (*β* = .26, *p* < .001) and disadaptive coping (*β* = –.43, *p* < .001) were also significantly associated with the mental component of the quality of life, while no association was found with adaptive coping (*β* = .09, *p* = .192). As can be observed, association of resilience with the mental component of quality of life was reduced comparing with the previous analysis with mental health, and conversely, association between disadaptive strategies and the output variable was slightly increased. Thirdly, when analysing the influence of predictor variables over the physical component of quality of life, only statistically significant and positive association was found with resilience (*β* = .20, *p* = .024).

**Table 3:** Hierarchical regression analysis of resilience and coping strategies over health.

	GHQ			SF-M			SF-F		
	*β*	*t*	*p*	*β*	*t*	*p*	*β*	*t*	*p*
**Resilience**	-.45	-7.012	.001	.26	3.585	.001	.20	2.276	.024
**Adaptive cop**	-.07	-1.199	.232	.09	1.311	.192	-.06	-0.718	.474
**Disadapt cop**	.38	6.282	.001	-.43	-6.284	.001	-.10	-1.168	.244
R^2^	.520			.381			.063		
*F*	59.69			33.61			3.67		
*p*	< .001			< .001			.014		

**Note**. β = beta coefficient; *t* = t-Student; *p* = level of significance; ⋀R^2^ = increase of explained variance; R^2^ = coefficient of determination; *F* = F of Snedecor.

### Structural model in the oncological sample

**Fig 1:**
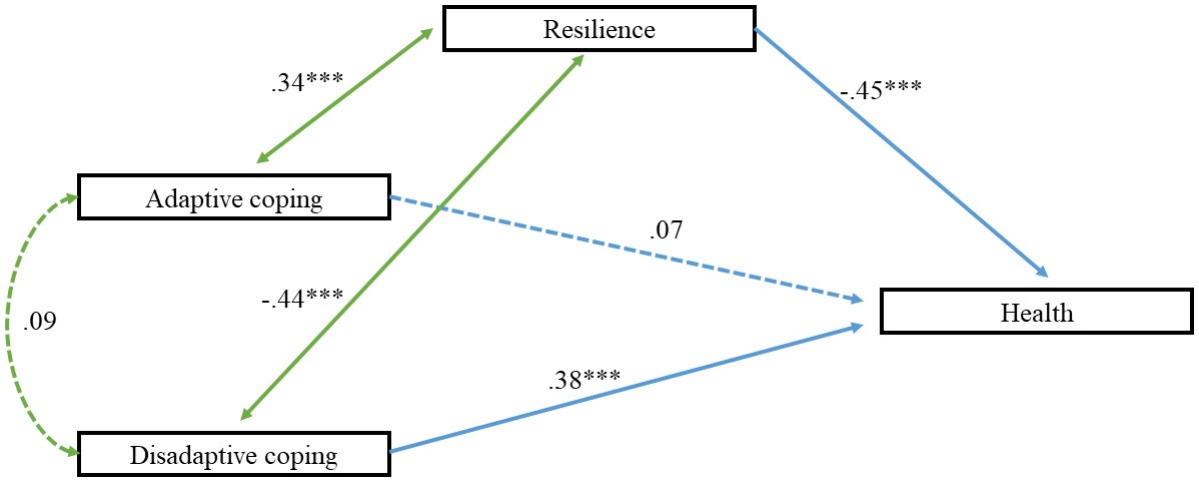
Model of relations between variable of resilience and coping strategies regarding health indicators. *** = significant.

Fig 1 illustrates the final model in the oncological sample. Almost all the factor weights were between.35 and.45 in health, resilience and coping; except from some dimensions that presented no statistically significant values. The model showed that adaptive coping was related to resilience (*β* = .34), which was directly linked in a negative way to health in people with cancer (*β* = –.45). However, adaptive coping did not show any relationship with disadaptive coping or health, reflecting no statistically significant results. Regarding disadaptive coping strategies, they were negatively and significantly correlated with resilience (*β* = –.44), while they showed a positive association with health *(β* = .38).

Furthermore, indexes of the model conducted in this study are shown, corrected from the data obtained. The Wald test suggested the elimination of the relationship between adaptive and disadaptive coping strategies (*β* = .09), and also the relationship between adaptive coping and health (*β* = .07). The indices obtained reflected an adequate fit (*χ*^2^_(70)_ = 2.399; *p* = .301, *CFI* = .99, *GFI* = .99, *AGFI* = .96, *SRMR* = .046, *RMSEA* = .034 [.000, .161]). The measurement model fitted, and all the variables were statistically significant (*t* >1.96).

## Discussion

Considering the significance of psychological variables on health-related processes (regarding quality of life, adherence to treatments, attending screenings, improving well-being…) [36], the aim of this study was to analyse relationships between coping strategies, resilience and health in a sample of people with cancer. Further, this study aimed to explore the underlying effect of psychosocial variables on cancer patients´ health.

Firstly, results showed that resilience and both types of coping strategies were significantly associated. Adaptive coping was positively related to resilience, while disadaptive coping was negatively linked to resilience. These findings are consistent with other studies that have found that adaptive coping (specifically, problem-focused coping, through strategies such as self-efficacy coping and/or acceptance) is related to potential protective factors in psychological well-being and mental health in people with cancer [37, 38]. On the contrary, a more disadaptive coping (for example, through strategies such as rumination, suppression and self-blame) has been related to potential risks of distress in people with advanced cancer [4, 11, 39]. These results have significance, as a greater use of adaptive coping strategies may involve a major empowerment of the person and a more active coping when facing stressful and difficult situations, as is cancer disease [40]. Hence, these findings imply that effective and adaptive coping strategies and resilience are associated with the maintenance of psychological adjustment in cancer patients, despite the oncological treatment and the negative consequences of the disease. Other studies support significant effects of the relation between coping strategies and resilience in cancer patients´ health status. A previous study conducted with 74 cancer patients explored how different levels of resilience (low, medium and high) derived in different responses to adaptation to disease. Patients with higher level of resilience showed greater use of adaptive coping (mainly, strategies of acceptance and positive revaluation) and better perception of quality of life. However, patients who showed lower level of resilience reflect significantly lower quality of life perception, with remarked differences in the dimensions of pain and general health, in comparison with the patients with higher resilience [41].

Secondly, results reflected an absence of significant correlation between adaptive and disadaptive coping strategies. These results support the existence of differences between both types of coping in cancer patients [42]. A person who presents a more adaptive coping tries to deal with the adversity and to modify the uncomfortable situation, in order to adapt to the disease [10]. On the contrary, disadaptive coping strategies could lead to a greater psychological distress (with higher levels of anxiety and depressive symptoms), a decrease in the psychical and psychological quality of life, more symptoms related to the disease such as fatigue or a worse sleep quality, even a worse adherence to the oncological treatment [18].

Thirdly, regarding to the relationship between resilience and coping strategies regarding health outcomes, results in this study showed that resilience was the factor that most correlated with level of health in cancer patients. Resilience was negatively associated with it, which means greater level of health. However, disadaptive coping strategies were negatively and significantly linked to health, which means a lower level of health.

As can be observed, psychological adaptation to the disease has been measured by means of an increase in the general level of general health. Results in this study are consistent with the idea that high levels of resilience contribute to improved psychological well-being and quality of life in people with cancer [20, 41, 43]. In this context, Min et al. [16] proved the protective effects of resilience on minimizing emotional distress in hospitalized cancer patients. Some authors have investigated which factors contribute to increase levels of resilience, suggesting that resilient people might be characterized by specific features including self-reflection, high responsibility, tolerance to negative feelings, etc. It seems that interactions between genetic, developmental, biological and psychosocial aspects determine differences in resilience [44]. However, it has also been considered as a dynamic process, which is susceptible to be modified by adequate interventions [15, 16].

### Study limitations

The study has focused on people with cancer who have been diagnosed by different types of tumours, so results may not be generalizable to other cancer populations. Besides, findings may be limited to be generalized to other stages or times throughout the process of the disease (diagnosis, later survivorship…). For further studies, it would be advisable to try to increase the sample homogeneity regarding these clinical variables. Furthermore, regarding the model obtained in this study, it should be considered that it is based in a cross-sectional study, which could have implications for the findings. In future research, it would be interesting to conduct a longitudinal analysis in order to explore the predictive effects of the principal variables on health outcomes.

This study has been limited to the assessment of specific psychosocial variables: resilience and coping strategies. In this respect, it would be adequate to introduce other variables´ measurement for future research, such social support, self-control, etc. in people with cancer. Furthermore, results obtained in this study regarding mental health should be deeply explored and confirmed in further studies, for instance, assessing depression, anxiety and stress with specific measures.

Another line of future research would be to elucidate if resilience is a static feature of the person or if it is a dynamic process [15]. It would be useful to consider it for future applications in psycho-oncology. It also would be appropriate to expand on this research by exploring if varying levels of resilience generate differences in coping patterns in people with cancer.

### Clinical implications

Given the importance of adaptation to oncological disease process, it is important to encourage an adequate coping and a good adjustment. While assessment of predictive risk variables aids to identify distressed patients, it is important to acquire knowledge about the variables that generate a positive impact of cancer patients´ level of health, with the aim of guiding clinical interventions [45, 46]. The current research highlights the importance of increasing adaptive coping and resilience to improve mental health and quality of life. In this sense, treatment options with cancer patients have been limited and they have been more commonly focused on palliative care [47]. Psychosocial interventions could be helpful to reduce the risks of emotional distress and improve mental health of patients at any stage of the disease [15, 39]. It is about providing guidance and advice to patients, with the goal of empowering them and helping to raise awareness of dealing with the disease in an effective way. Additionally, given that cancer patients could benefit from psychological interventions, this service should be offered to participants in order to provide an adequate clinical care.

## Conclusion

To summarise, this study has provided information about the role of resilience and coping strategies in cancer patients´ mental health and quality of life through a cross-sectional design. Findings in this research underscore the positive contribution of high levels of resilience and an adaptive coping on participants´ level of general health. On the other hand, disadaptive coping strategies did not reflect any positive relation to resilience or health indicators. Thus, promoting resilience and adaptive coping could be a significant goal for psychosocial and educational interventions in people with cancer.
